# Preservation of organic matter in marine sediments by inner-sphere interactions with reactive iron

**DOI:** 10.1038/s41598-017-00494-0

**Published:** 2017-03-23

**Authors:** Andrew Barber, Jay Brandes, Alessandra Leri, Karine Lalonde, Kathryn Balind, Sue Wirick, Jian Wang, Yves Gélinas

**Affiliations:** 1 GEOTOP and the Department of Chemistry and Biochemistry, Concordia University, 7141 Sherbrooke West, Montréal, Quebec, H4B 1R6 Canada; 2 Skidaway Institute of Oceanography, University of Georgia, 10 Ocean Science Circle, Savannah, GA 31411 USA; 3 Department of Natural Sciences, Marymount Manhattan College, 221 E 71st St., New York, New York 10021 USA; 4 National Synchrotron Light Source, Brookhaven National Laboratory, Upton, NY 11973 USA; 5 Canadian Light Source Inc, Saskatoon, Saskatchewan S7N 0X4 Canada

## Abstract

Interactions between organic matter and mineral matrices are critical to the preservation of soil and sediment organic matter. In addition to clay minerals, Fe(III) oxides particles have recently been shown to be responsible for the protection and burial of a large fraction of sedimentary organic carbon (OC). Through a combination of synchrotron X-ray techniques and high-resolution images of intact sediment particles, we assessed the mechanism of interaction between OC and iron, as well as the composition of organic matter co-localized with ferric iron. We present scanning transmission x-ray microscopy images at the Fe L_3_ and C K_1_ edges showing that the organic matter co-localized with Fe(III) consists primarily of C=C, C=O and C-OH functional groups. Coupling the co-localization results to iron K-edge X-ray absorption spectroscopy fitting results allowed to quantify the relative contribution of OC-complexed Fe to the total sediment iron and reactive iron pools, showing that 25–62% of total reactive iron is directly associated to OC through inner-sphere complexation in coastal sediments, as much as four times more than in low OC deep sea sediments. Direct inner-sphere complexation between OC and iron oxides (Fe-O-C) is responsible for transferring a large quantity of reduced OC to the sedimentary sink, which could otherwise be oxidized back to CO_2_.

## Introduction

As the largest sink for organic carbon (OC) on Earth, marine sediments play a major role in the global carbon cycle^1^. The majority of the OC preserved within sediments is intimately associated to the mineral matrix through sorption on clay minerals and metal oxides^1–5^. In particular, redox sensitive, nano-scale iron oxides have a strong affinity for OC^6^, forming stable Fe-OC complexes that can persist for thousands of years in anoxic sediments at depths of up to 5 m^7^. These high surface area-to-volume ratio particles readily bind OC and increase its stability, transport and sequestration in sediments^6, 8^. As such, reactive iron oxides (defined as dithionite reducible iron oxides) constitute a “rusty sink” that accounts for the preservation of 21.5 ± 8.6% of sediment OC in the global ocean^7^. These Fe-OC interactions must be practically irreversible under natural conditions to account for the long-term preservation of reactive OC^9^ since reversible binding (i.e., cation bridging, hydrogen bonding, van der Waals and hydrophobic interactions) eventually leads to the solubilisation of OC, ultimately resulting in its enzymatic hydrolysis and biodegradation^10, 11^.

The interactions between Fe and OC in natural settings have been extensively studied in the past, but most research has been conducted using model OC compounds and preformed iron oxides in experiments that cannot fully mimic sediment redox boundaries/microenvironments, formation conditions, the timescales of carbon cycling or the diversity of species participating in these interactions. Iron oxides, for example, can be detrital (preformed lithogenic particles sedimenting from the water column) or authigenic (formed *in-situ* through the oxidation of pore water Fe^2+^ in the presence of OC). Complexation of OC and iron oxides at the sediment redox boundary can result in strong inner-sphere interactions^12^, poisoning the crystal structure of iron oxides^13^, while also stabilizing reactive OC. These strong inner-sphere Fe-O-C chemical bonds, also referred to as covalent interactions, act in consort with others sedimentary mechanisms (i.e., sorption by clays, geopolymerisation, intrinsic recalcitrance, and physical protection by biominerals^1^) to transfer large quantities of reduced OC from the active surface of the globe to its slowly cycling interior where it remains locked on geological time scales.

Sequential extraction methods used to probe iron-OC interactions involve harsh chemical treatments that target operationally defined iron fractions^14–16^, with the OC:Fe molar ratio of the extracted material being used to infer molecular-level interaction mechanisms^7, 17^. The use of ratios however assumes that the entire iron pool is bound to OC, neglecting the possibility that only a fraction of the extracted reactive Fe is associated with OC. While measuring the percentage of total OC associated to Fe is straightforward^7^, obtaining the percentage of total reactive Fe directly complexed to OC is more challenging.

Here we use Fe K-edge X-ray Absorption Near Edge Structure (XANES) spectroscopy to quantify the proportion of reactive Fe involved in inner-sphere complexation to OC in contrasting sedimentary environments. In conjunction with the XANES spectroscopy, key organic functional groups co-localized with ferric iron were identified using X-ray spectromicroscopy at the C K-edge and Fe L_3_-edge. We probed for the first time the actual preservative interactions occurring between iron and OC in chemically unaltered sediments.

## Materials and Methods

### Samples

The samples comprise sediments of different composition, accumulating in contrasting depositional regimes under varying redox conditions (Table 1). They include highly oxidized, OC poor (<0.33 wt%) pelagic sediments (Equatorial Pacific red clays at 9**°**N, Equatorial Pacific carbonates at 0**°**N, an opal-rich sediment from the Southern Ocean, and a highly oxidized turbidite from the Madeira Abyssal Plain off the Moroccan Coast – MAP2). Also included are a series of coastal samples accumulating under varying redox conditions: Arctic Margin sediments from oxygenated bottom waters close to the Mackenzie River delta, Mexican Margin sediments accumulating under oxic (station 305) and suboxic (station 306) bottom waters conditions offshore from the city of Mazatlan, sediments underlying the perennially hypoxic bottom waters of the St. Lawrence Estuary (Quebec, Canada), oxic sediments collected in the Arabian Sea off the coast of Oman, the seasonally anoxic Saanich Inlet (British Columbia, Canada), and the sulfidic Black Sea samples.

**Table 1:** Composition of the samples and raw sediment Fe K-edge Linear combination fitting results.

Sediment	Depth (cm)	Total OC content (wt%)	C/N^(a)^	δ^13^C (‰)	Sediment total Fe content (mg Fe/g sed)	Free iron oxide contribution to total sediment Fe (wt%)	Iron-OM complex contribution to total sediment Fe (wt%)	Residual non-reactive Fe contribution to total sediment Fe (wt%)	Sum of all fitting components (%)	OC bound to Fe^(b)^ (% of total OC)
Saanich Inlet	0–20	2.21	7.51	−22.4	23.1	10.8 ± 2.2^(c)^	18.1 ± 2.6	72.0 ± 4.9	101	28.09
Arabian Sea	0–0.5	1.11	8.04	−21.6	17.3	26.5 ± 7.1	16.6 ± 6.3	57.4 ± 1.6	101	26.89
Madeira Abyssal Plain turbidite	119–121	0.29	15.04	−21.9	39.8	68.6 ± 5.7	12.5 ± 6.1	11.7 ± 1.6	93	NA
Black Sea	0–0.5	4.61	15.69	−26.6	33.3	0.0 ± 3.7	8.1 ± 1.0	90.4 ± 3.4	99	24.98
Mexican Margin (Station 306)	0–0.5	6.66	9.27	−22.1	30.7	15.1 ± 1.9	7.8 ± 0.7	74.6 ± 2.3	98	22.31
Mexican Margin (Station 305)	0–0.5	2.82	9.00	−21.5	35.4	19.4 ± 5.0	6.7 ± 0.6	76.0 ± 2.2	102	12.70
Southern Ocean	8–12	0.33	8.27	−20.8	7.7	14.8 ± 4.9	5.3 ± 0.9	82.2 ± 3.9	102	29.00
St. Lawrence Estuary	0–35	1.43	12.83	−24.3	32.6	12.8 ± 0.4	5.1 ± 1.3	80.6 ± 1.6	99	25.13
Equatorial Pacific 0°N	0–0.5	0.27	8.26	−21.6	3.2	40.4 ± 5.1	—^(d)^	61.1 ± 1.1	102	34.79
Arctic Margin	0–1	1.18	7.35	−26.2	50.9	41.1 ± 3.5	—	56.9 ± 1.3	98	7.60
Equatorial Pacific 9°N	10–12	0.30	4.58	−22.4	35.7	13.9 ± 2.0	—	87.6 ± 4.6	102	12.16

^(a)^Atomic C/N ratio.

^(b)^Percent of total sediment OC associated to reducible iron oxides taken from ref. 7.

^(c)^1σ errors for the fitting results.

^(d)^Component not used in the combinatorial linear fitting calculations.

Sediments from oxygen limited (Saanich Inlet and Mexican Margin) and sulfidic environments (Black Sea) have higher OC content compared to the other sediments included in this study (Table 1). Of particular interest to this study is the nature of the organic matter which is deposited in these sediments with the Saanich and Mexican Margin samples being dominated by mostly marine inputs while the Black Sea is dominated by terrestrial organic inputs, as shown by the their higher atomic C/N ratio and more depleted δ^13^C signatures (Table 1). The remaining coastal sediment samples were chosen to represent a wide range of depositional settings along the terrestrial-marine continuum.

### Sediment Organic Carbon Content Determination

Sediments were first decarbonated overnight in an acid (12N HCl) fumigation chamber, followed by gentle drying for 1 hour. The organic carbon content of the decarbonated sediment was determined using a CHN analyzer (EuroVector 3028-HT) using β-alanine (40.4% OC) as a calibration standard.

### Synthetic Fe hydroxides and OC:Fe Complexes

Ferric oxalate (C_6_Fe_2_O_12_), purchased from Sigma-Aldrich Canada, was used as an inner-sphere OC-substituted Fe(III) model compound. Lepidocrocite (γ-FeO(OH)), an OC-free ferric hydroxide, was synthesized following the procedure of Cornell and Schwertmann^18^. Briefly, ferrous chloride was added in solution and precipitated out as ferric hydroxide through the dropwise addition of 1 M NaOH, maintaining circumneutral pH, while continuously bubbling O_2_ through the solution. Precipitation of iron hydroxides was also performed in the presence of varying concentrations of dissolved algal organic matter prepared by repeated freeze/thaw cycles of *Nannochloropsis* cells (Reed Mariculture Inc., CA, USA) in liquid nitrogen, liberating a highly concentrated dissolved OM solution which was filtered using a 0.7-μm glass fiber filter in the same fashion as^19^. While dissolved OM derived from fresh plankton cells might differ from pore water dissolved OM where precipitation of natural OC:Fe takes place, it was used only in this proof-of-concept experiment to show the effect of OM complexation to the XANES Fe spectrum (see below). The quantity of dissolved OM was adjusted in order to have initial OC:Fe molar ratios in solution of 1, 3 and 10. The precipitation reaction was performed by oxidizing ferrous chloride in the dissolved algal OM solution, maintaining a circumneutral pH through dropwise addition of 1 M NaOH, in the same fashion as for the precipitation of lepidocrocite. These synthetic solid phases were transferred to Kapton tape for the Fe K-edge X-ray analyses. In addition to synthetic lepidocrocite, we also analyzed commercial goethite (Sigma-Aldrich), but the acquired Fe K-edge XANES spectrum for goethite was indistinguishable from that of lepidocrocite, as also reported in a previous study^20^.

### Fe Mass Balance

The Fe present in each sample was apportioned into three different components: first, the reducible (henceforth called “reactive”) iron oxide fraction was determined using the dithionite-citrate-bicarbonate extraction method of Lalonde *et al*.^7^. The Fe concentration in the extract was measured by ICP-MS following acidification to pH < 2 with 16 N HNO_3_. The reactive iron-free residue was then digested by gentle heating with the addition HCl (12 N), HNO_3_ (16 N) and HF (28 N). Following evaporation to near-dryness, the final residue was dissolved in a 10% HNO_3_ solution and its iron content was analyzed by ICP-MS. Finally, the XANES spectra acquired for the natural (non-extracted) sediments and their reactive iron-extracted counterpart further allowed quantifying, within the reactive iron fraction, the proportions of OC-free iron oxides and iron oxides associated to OM using a linear combinatorial approach (see below).

### Iron X-ray Absorption Near Edge Structure Spectroscopy

XANES and NEXAFS (Near Edge X-ray Absorption Fine Structure) spectroscopy target the same region of the X-ray absorption spectra but the two terms are used separately in this text to distinguish between the results for the hard and soft X-ray experiments, respectively. Iron K-edge XANES spectra were collected at beamline X26A at the National Synchrotron Light Source (BNL, NY, USA) using an Si(111) monochromator. To minimize beam damage, the beam was slightly de-focused, giving a spot size of 50 µm^2^_._ Each end-member used for spectral deconvolution was the averaged XANES spectra from at least 3 different locations on the sample. Two or three replicate X-ray spectra for the raw sediment samples were collected and averaged prior to spectral deconvolution. The beamline energy was calibrated by repeated measurements of an iron foil with a known K-edge absorption band at 7112.0 eV and a nontronite (Fe-rich smectite) standard was run intermittently throughout the runs to account for any energy drift during the data acquisition. The XANES spectra were normalized using a polynomial fit in order to compare samples of different thicknesses and Fe content, and finally background corrected using the built-in Rbkg algorithm prior to the linear combination fitting. All Fe K-edge data reduction was performed using the ATHENA software package^21^.

### Iron K-edge XANES Linear Combination Fitting

**Figure 1:**
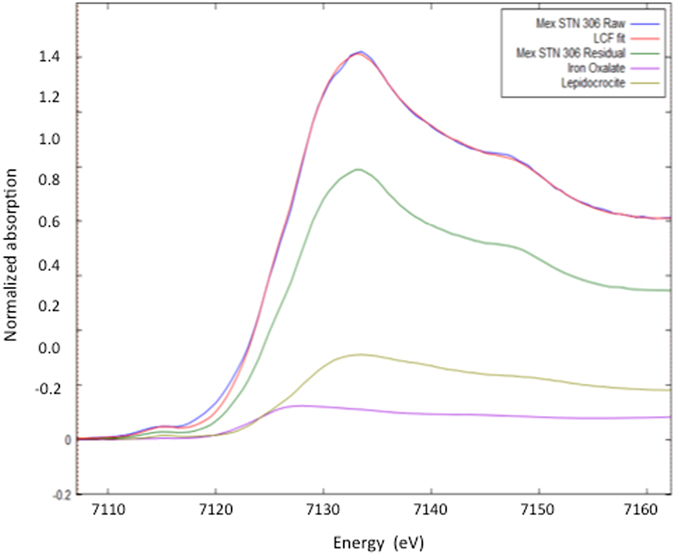
Iron K-edge XANES Linear Combination Fitting of the raw sediment Fe spectra for the Mexican 306 sediment using three end-members: goethite/lepidocrocite, ferric oxalate and the reactive iron extracted sediment residue. The best fit (red trace) is almost superimposed on the spectra for the raw, untreated sediment (blue trace).

Raw sediment Fe spectra were fit using a linear combination of iron XANES spectra from selected iron reference compounds and treated sediments. The fitting was performed from 20 eV below E_0_ to 35 eV above E_0_, without forcing the sum of each component to be equal to 1. The sum of all fitting components for each sample can be found in Table 1. Each raw sediment spectrum was fitted in a combinatorial fashion using the XANES spectra for the following three end-members: goethite/lepidocrocite, ferric oxalate and the reactive iron extracted sediment residue. The best fits were selected to match the experimentally determined proportion of Fe remaining after the Dithionite-Citrate-Bicarbonate (DCB) treatment with the spectral deconvolution results (Fig. 1). In all cases, the selected fit had a Fe percent contribution for the residual sediment within error from the actual measured Fe content. A similar approach using *a priori* knowledge of a sample to simplify complex systems has been exploited before^22, 23^, although not by using a post extraction residue as one of the end-members. The 1σ errors for each fitting component can be found in Table 1 and represent the error associated to using model compounds in order to deconvolute the XANES spectra of natural samples from complex biogeochemical cycle such as the sediment iron cycle.

### STXM Sample Preparation and Mapping

Freeze-dried, homogenized sediment samples were embedded in an elemental sulfur resin in place of the typical epoxy resin workup, to minimize sample oxidation. A 1:5 ratio of sediment to sulfur was heated to ~140 °C, and allowed to cool/solidify. The solidified sediment sulfur mixture was then sliced using an ultra-microtome to a thickness of 80 to 120 nm and plated on a Transmission Electron Microscopy (TEM) grid for analysis at the beamline.

**Figure 2:**
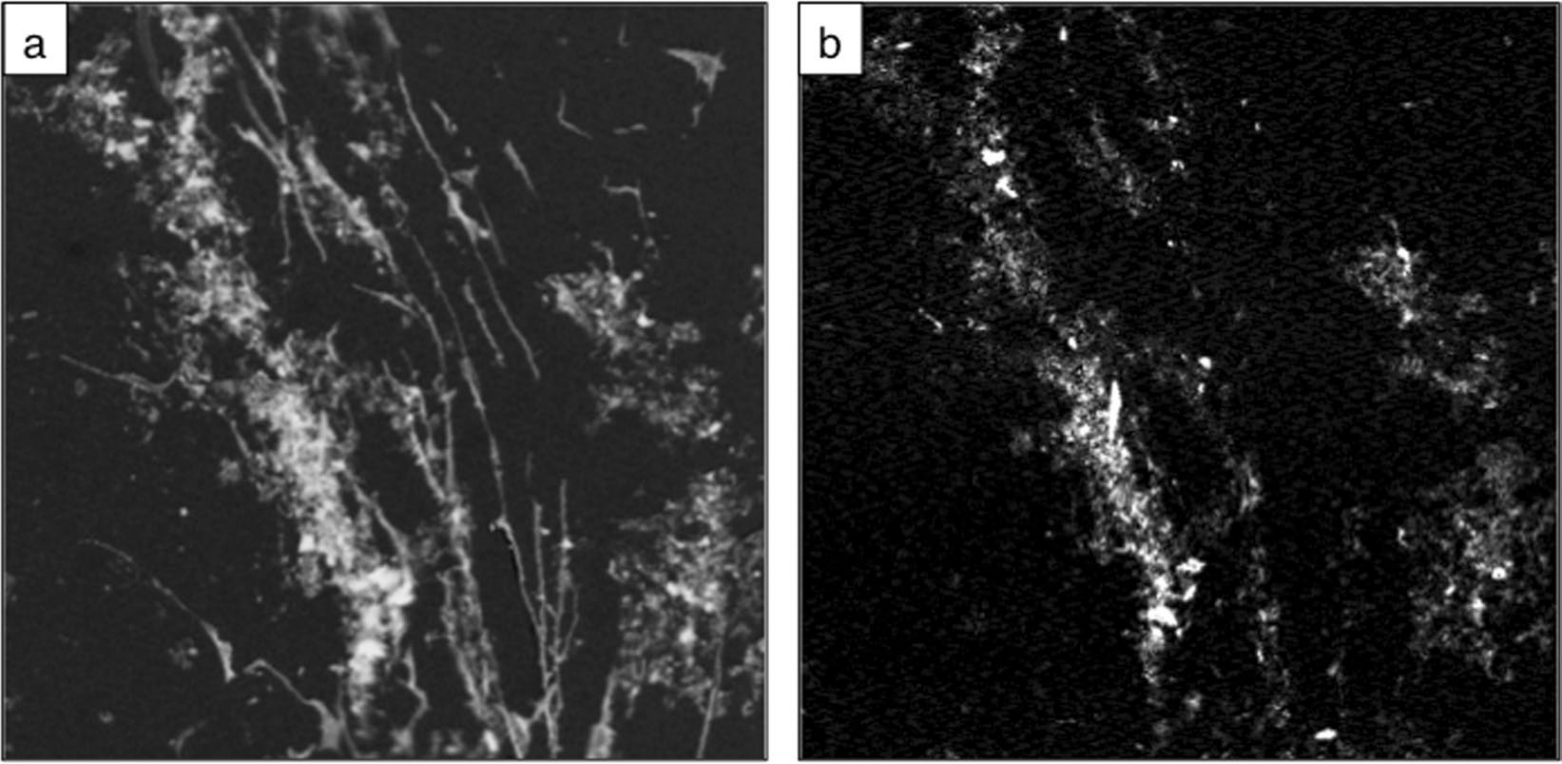
STXM “Scan Maps” collected between (**a**) 280 and 288.6 eV for carbon, and (**b**) between 700 and 710 eV for iron, respectively. These maps allowed selecting areas where OC and Fe were co-localized in our samples for NEXAFS collection.

Initial X-ray “Scan Maps” were collected over a larger portion of the sample at 280 and 288.6 eV as well as 700 and 710 eV for C and Fe determination, respectively. After normalization to the intensity at the empty areas of the measured sample region, the pre-edge images collected at 280 eV and 700 eV were subtracted from the images collected at 288.6 and 710.0 eV (Fig. 2), which correspond to absorption bands for carbonyl and the dominant Fe L_3_ edge peak for ferric minerals, respectively. These species are ubiquitous in marine sediments and were thus used as indicators for the presence or absence of OC and Fe in these samples. From these images we selected regions where OC was found co-localized to Fe (found in all samples) and regions where OC was not found to be co-localized to Fe (when possible) in order to compare Fe-associated and Fe-free organic functionalities.

### Near Edge X-ray Absorption Fine Structure Spectroscopy

C and Fe NEXAFS image stacks were collected from 280–320 eV and 700–735 eV. Despite the nano-scale thickness of the samples, several regions showing co-localization of OC to Fe were characterized by a dip centered around 284 eV in the spectrum and a secondary, less pronounced dip around 290 eV. These dips likely are caused by a loss in signal intensity arising from contaminant organic matter present on the mirrors and lenses of the beamline, absorbing a portion of the incoming beam flux. High OC content samples did not show this feature. The presence of these dips was minimized using ultra-thin sliced samples (80–120 nm). Note that for the Madeira Abyssal Plain turbidite sample, the absolute amount of C was too low to allow acquiring useful NEXAFS spectra.

NEXAFS image stacks were collected every 40–60 nm over the regions of interest. The image stacks were aligned along the X and Y axis, using each preceding image and the “Stack Analyze” software from^24^. The X-ray image stacks were simplified using the PCA GUI software^25, 26^ in order to cluster the data into regions with similar spectral properties. This was done first by normalizing to the background (I_0_) X-ray spectrum from the previously aligned stacks of spectra, then running a cluster analysis following the procedure of Lerotic *et al*.^26^.

### Redox Calculations

A rough assessment of the burial of reducing power stemming from the protection of OM through OC:Fe interactions was calculated from our dataset, using the following redox data. First, the average oxidation state of marine OC was calculated to be −0.48 based on the average elemental composition of fresh planktonic OM (C_106_H_177_O_37_N_17_S_0.4_), as determined by Nuclear Magnetic Resonance (NMR)^27^. Since this marine OC is oxidized to CO_2_ (oxidation state of +4) when the organic matter is completely remineralized under oxic conditions, the complete oxidation of one mole of OC involves the transfer of 4.48 electrons to electron acceptor species such as dissolved O_2_. Note that the reduction of ferric iron to ferrous iron is mediated through the transfer of only one electron.

## Results and Discussion

### XANES Analyses

**Figure 3:**
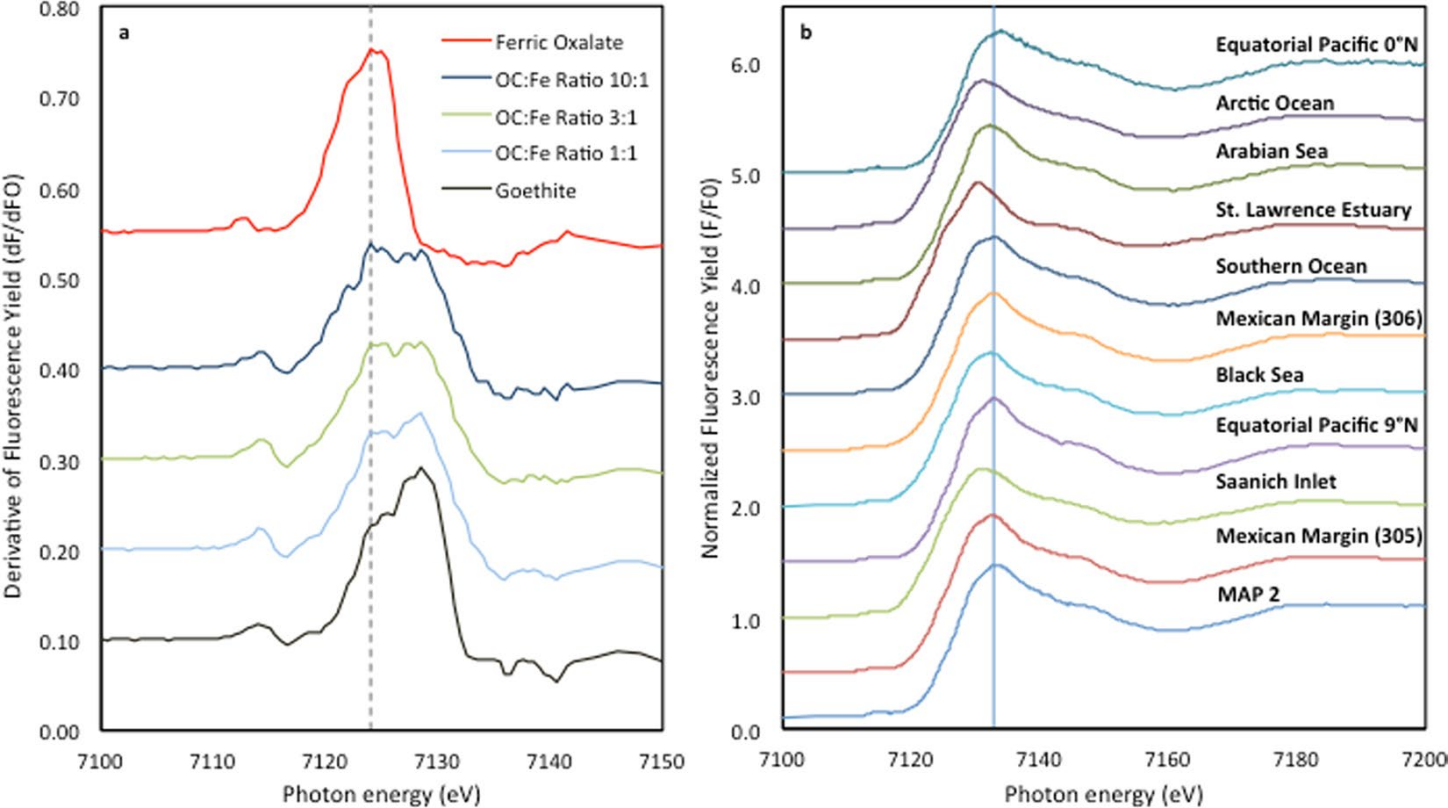
Iron K-edge spectra for the samples analyzed in this study. (**a**) First derivative of Fe 1*s* XANES spectra for goethite, ferric oxalate and synthetic lepidocrocite with initial solution OC:Fe Molar ratios of 1, 3, and 10. The intersecting line at 7124.5 eV highlights the shift in absorbance maxima for ferric oxalate; (**b**) Normalized Fe 1s XANES spectra for raw sediments with the vertical line at 7132.8 eV representing the absorption maximum for goethite/lepidocrocite.

Iron K-edge XANES spectra were collected for the pure iron materials (lepidocrocite and ferric oxalate) and the three synthetic Fe-OC complexes, precipitated from solutions with intial OC:Fe ratios of 1, 3 and 10, respectively (Fig. 3a). A key feature of these Fe spectra is the ~4 eV difference between the absorption maxima for pure iron oxides and ferric oxalate, an inner-sphere (Fe-O-C) complex whose coordination environment is dominated by σ and π covalent interactions^28^. The lower-energy feature was also observed for synthetic Fe-OC co-precipitates, with its relative intensity increasing when iron oxides were formed in the presence of more dissolved OC (Fig. 3a). This systematic increase in intensity of the low-energy Fe-OC feature allowed determining the proportion of iron complexed to OC through inner-sphere interactions using spectral deconvolution and linear combination fitting.

Iron K-edge XANES spectra were then collected for a series of sediments and for their residues following extraction of reactive iron oxides (Fig. 3b). These spectra were fitted using three end-members: (1) OC-free reactive iron oxides, (in-house synthesized lepidocrocite and commercial goethite from Sigma Aldrich); (2) inner-sphere OC-substituted Fe(III) (ferric oxalate); and (3) residual unreactive iron, corresponding to post reduction sediment residue from each individual sample, as several less reactive iron species not targeted by the reactive iron removal procedure, such as iron-containing silicates or iron sulfides, may be present in varying proportions in the residue. Such differences in composition and relative abundances of non-reactive Fe-bearing minerals would make the selection of an adequate synthetic end-member for non-reactive iron extremely challenging^29^.

### Iron data by XANES

**Figure 4:**
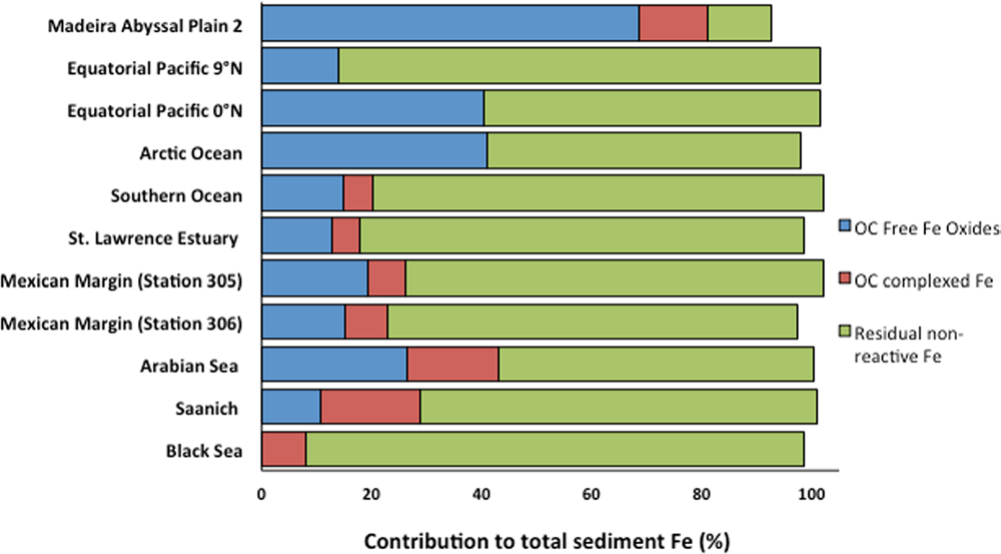
Iron K-edge XANES linear combination fitting results showing the OC-free iron oxides (blue), OC-complexed reactive iron (brown), and residual sediment iron (green) contributions to the total sediment Fe pool.

Linear combinatorial fitting calculations revealed that the bulk of the sedimentary iron (56.9 to 90.4%) is found in the residual non-reactive fraction, with the exception of the extensively oxidized MAP2 turbidite (11.7 ± 1.6%) (Fig. 4, Table 1). Total reactive iron, which is the sum of the relative contributions from OC-free iron oxides (Table 1 Column 6) and OC-complexed iron (Table 1, Column 7) to total Fe, accounts for 8.1 to 43.1% of total Fe, with variable proportions of OC-free iron oxides and OC-complexed iron. The MAP2 sample again is an outlier with 81.1 wt% of total iron found in the reactive iron pool. The relative contribution of OC-complexed Fe accounts for 0 to 18.1 wt% to total iron in the sample. The selected linear combination fitting results for the Equatorial Pacific and Arctic sediments did not include the OC-complexed Fe component, a sharp contrast to Black Sea where this component accounts for 100% of reactive Fe. Interestingly, the highest relative contribution of OC-bound Fe to total reactive Fe is found in the only sulfidic sediment (Black Sea). Although more work is needed to confirm this hypothesis, this result could be due to the fact that reactive iron oxides are not expected to survive in sulfidic environments unless stabilized by organic matter or other surface reactive compounds^13, 30^.

Conversely, the non-detectable contribution of the Fe-OC fraction in the Equatorial Pacific and Arctic samples can be explained by their very low OC content (~0.30 wt%) (Table 1) and high Fe content, respectively (Table 1). The OC-bound Fe contribution to total reactive iron in the coastal samples (Saanich, Arabian Sea, Mexican Margin and St. Lawrence Estuary) varies between 25.7 and 62.6%, supporting earlier results that highlighted the role of iron in the preservation of organic matter in coastal sediments^7^, where the bulk of the sedimentary OC pool is sequestered globally^1^. The high proportion of reactive Fe covalently bound to OC likely reflects the fact that these interactions take place both at the surface and within iron oxide particles or aggregates.

### Co-localization of OC and Fe by STXM

**Figure 5:**
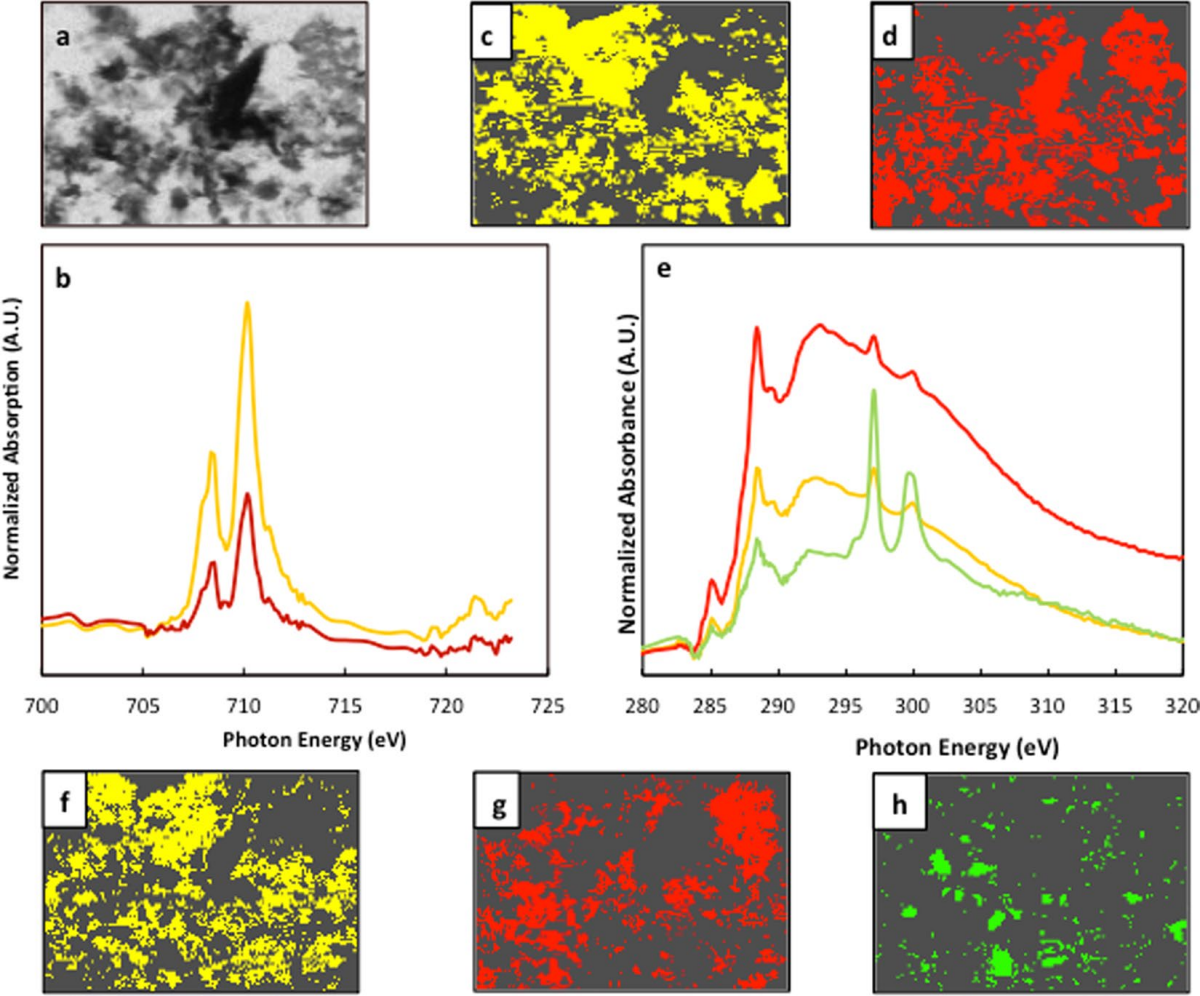
Chemical composition of co-localized iron (panels a and b–d) and organic matter (panels a and e–h). (**a**) Representative 12 × 8 μm X-ray microscopy image from the Mexican Margin sediment (Station 306); (**b**) Iron L_3_ edge XAS spectra for the Fe cluster maps in panels (**c**–**e**) Carbon K-edge XAS spectra for the (**c**) cluster maps in panels (**f**–**h**). Note that colored traces in panels (**b** and **e**) correspond to the different coloured carbon clusters in panels (**c,d** and **f–h**), respectively. Each cluster was identified using the PCA and clustering analysis described in the methods section.

Using Scanning Transmission X-ray Microscopy (STXM; Canadian Light Source beamline 10ID-1), a subset of the samples were analyzed to assess OC co-localization with iron, and to determine whether specific OC functionalities are preferentially co-localized with Fe oxides in sediments. Carbon K-edge and Fe L_3_-edge X-ray absorption spectra (XAS) coupled to x-ray microscopy images of the sulfur-embedded samples were collected at a 40-nm resolution (Fig. 5a). X-ray absorption image stacks were then acquired in OC- and Fe-rich regions allowing identification of the OC functionalities and determination of the oxidation state of Fe (Fig. 5b and e). The XAS for most OC co-localized with Fe is dominated by peaks around 285.3 eV (aromatic, alkene C=C) and 288.5–288.6 eV (carboxyl/carbonyl) in these sediments^31, 32^ (mapped areas similar spectral properties, or clusters, in Fig. 5f–h with corresponding traces in Fig. 5e). Another peak of interest at 289.5 eV suggests the presence of alcohols (C-OH^32^) co-occurring in most cases alongside aromatic and carbonyl functional groups. Overall, the absorption bands observed for these sediments are similar to those observed for complex soils and synthetic co-precipitates, with the notable absence of peaks at 286.6 eV commonly attributed to ketone^32–34^. Note that absorption bands in the post-edge region found at ~297 and 300 eV correspond to the potassium L-edge X-ray absorption bands^35^, but are not discussed further in this text.

The Fe L_3_-edge spectra provide information on the oxidation state of iron based on the relative intensities of two absorption bands (708 and 710 eV) representing the dominant absorption bands for ferrous and ferric iron respectively^32^. The majority of sediment OC was found co-localized to areas dominated by Fe(III) phases (Fig. 5b; in this figure, both traces show predominant Fe(III) phases, with a lower intensity peak corresponding to Fe(II)). Across all analyzed sediments, ranging from highly oxidized to fully anoxic environments, the co-localized OC functionalities correspond to chemical groups (carbonyls, alcohols, alkenes and aromatics) that have a high affinity iron oxides^36^, and are similar to those reported for OC-rich soils^32, 37^. Although co-localization does not necessarily reflect direct inner-sphere complexation, the Fe K-edge deconvolution data presented in this study, as well as several publications (reviewed in ref. 12) provide strong evidence for direct inner-sphere complexation between organic matter and iron in marine sediments. The presence of these ubiquitous functionalities raises interesting questions regarding the factors that promote the complexation of OC with reactive iron oxides. One of the most important is the affinity of carbonyl- and hydroxyl-containing aromatic acids, which bind to iron (oxy) hydroxides via a ligand exchange mechanism^36^, with organic matter displacing the hydroxyl surfaces from the reactive iron species. The mixed σ and π bond characteristic of these organic compounds readily allows for the formation of a highly stable OC:Fe complexes, similar to the interactions of iron with siderophores.

### Implications

Our findings have important implications with respect to the global Fe and OC cycles, with ramifications that extend to the marine sediment organic carbon sink and global climate change. First, while the nano-scale co-localization of sediment OC and Fe has been reported before, we show for the first time the importance of direct inner-sphere complexation involving aromatic, carbonyl and alcohol groups in unaltered natural samples (Fig. 5). We also provide the first direct assessment of the percentage of reactive Fe involved in strong Fe-O-C bonds, and find that between 25.7 and 62.6% of reactive iron is directly bound to OC in coastal settings, emphasizing the importance of Fe-OC interactions not only for the OC cycle but also for the Fe cycle.

**Figure 6:**
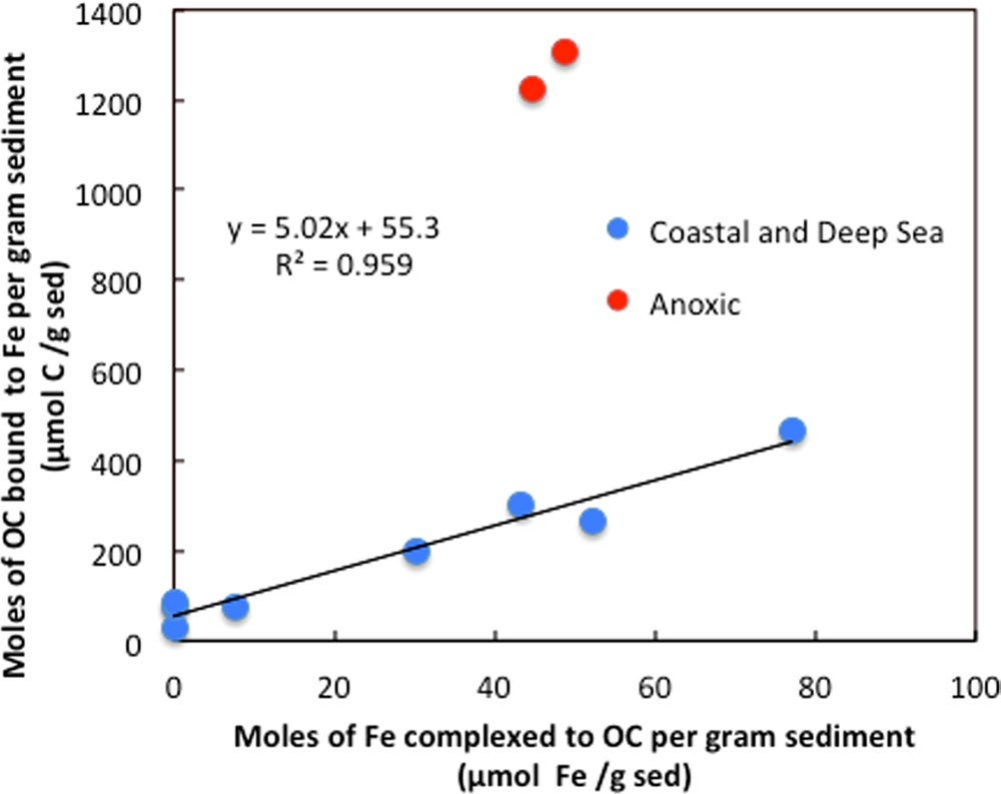
Correlation between the quantity of OC associated to iron oxides measured by wet chemical extraction^7^, and the quantity of Fe found complexed to OC measured by XANES (this work) for the samples studied in this work. Sediments accumulating under anoxic conditions are excluded from the regression (see text for explanations).

The fact that only a fraction of reactive iron is covalently bound to OC suggests that using OC:Fe ratios to infer binding mechanism^7, 17, 38^ should be avoided, as a quantitatively important fraction of reactive iron is not complexed to OC through inner-sphere interactions. For the coastal and deep-sea sediments studied here (excluding the Black Sea and Saanich Inlet sediments accumulating under anoxic conditions; see below), a strong linear trend was observed between the moles of OC associated to iron oxides per gram of sediment, measured following wet chemical extraction (from ref. 7), and the moles of reactive iron complexed to OC per gram of sediment, measured by XANES (this work; Fig. 6). The slope of ~5 observed in Fig. 6 represents the molar ratio of Fe-complexed OC to OC-complexed Fe (OC:Fe molar ratio). OC:Fe ratios have previously been used to infer bonding mechanisms between OC and Fe with OC:Fe ratios of ~1 indicating simple mono-layer sorption, while higher OC:Fe ratios being indicative of coprecipitation^38^. When determined using chemical extractions, the amount of OC-complexed Fe is overestimated as all reactive iron is extracted (OC-free iron oxides and OC-complexed iron). By providing an estimate of the fraction of reactive iron that is complexed to OC, the linear combination fitting results allow to correct for this bias. The slope of ~5 from Fig. 6 thus represents a more accurate estimate of the OC-to-Fe molar ratio characteristic of these inner-sphere complexes and agrees with the existence of agglomerates containing iron that is interweaved between layers of organic molecules, analogous to the onion model of Mackey and Zirino^39^, as opposed to the monolayer sorption hypothesis in which OC:Fe ratios of ~1 are expected.

The two sediments accumulating under anoxic conditions gave OC:Fe ratios of about 30, a value that suggests the existence of other OC stabilization mechanism(s) in which Fe is involved. Alternatively, such high ratios might also reflect the fact that large organic biomolecules may be only partly associated to iron oxides through only one or a few functional groups, a hypothesis that is more plausible under anoxic conditions (see below). The organic functionalities observed from the C K_1s_ X-ray absorption experiments could be indicative of large aromatic acids complexing Fe(III) via a ligand exchange mechanism. In such complexes only a fraction of the carbon atoms would be directly bound to Fe but would still allow for the OC:Fe molar ratios observed in the reducing environments studied here. Notably, these sediments also have much higher OC surface loadings (mg OC per square meter of surface area) compared to normal coastal sediments^40^. More work is needed to explain these high OC:Fe ratios.

Second, XANES analysis and spectral deconvolution show that the proportion of reactive Fe complexed to OC increases with decreasing oxygen exposure. The fraction of OC-complexed Fe negligible in low OC, highly oxidized deep-sea sediments and accounts for 100% of the reactive iron pool in sulfidic black sea sediments. The same relative enrichment in OC-complexed Fe was found in Fe-bearing minerals in soils exposed to prolonged regular alternation of redox conditions^41^. The irreversible nature of binding is particularly important for OC preservation in anoxic sediments where biological degradation first proceeds through solubilisation followed by enzymatic hydrolysis^42^. Anoxic respiration is less efficient and pervasive than oxic degradation in which reactive oxygen species lead to extensive and non-specific OC oxidation^43^. In such oxic environments, only physically shielded, inaccessible OC is efficiently preserved on a long-term basis^44^, partly explaining the very low OC concentrations and accumulation rates measured in pelagic settings.

Physical shielding of OC by iron oxides is best achieved in redox transition zones within sediments where dissolved Fe(II), a product of the reduction of iron oxides in the deep, anoxic sediment layers, diffuses upwards towards the oxic-to-anoxic sediment transition layer. Upon coming into contact with dissolved O_2_, which diffuses downward into the sediment from the water column, Fe(II) is oxidized and precipitates as Fe(III) oxides in the presence of dissolved or colloidal porewater OC, forming mixed mineral-organic co-precipitates aggregating on the surface of other mineral particles^45^. The degradation rate of OC complexed to Fe within these aggregates is much slower than those of dissolved or reversibly sorbed OC^3^.

Co-precipitation of Fe-OC aggregates also influences the fate of iron oxides in sediments. Free iron oxides are not thermodynamically stable under anoxic conditions and are thus reduced to soluble Fe(II). Complexation with OC physically protects iron oxides, sterically inhibiting their enzymatic and reductive dissolution, likely explaining the persistence of metastable reactive Fe(III) on very long time scales in reduced sediments (as long as 400 kyrs^30^). Although more work is needed to confirm this hypothesis, our interpretation agrees with the lack of organic-free iron oxides as a significant contributor to total Fe in the Black Sea sediment. This mechanism is analogous to a synergistic transport mechanism, or synergistic ferric OC shuttle, which, in conjunction with other OC preservation pathways, protects OC during its critical passage through the sediment oxic layers and facilitates its transfer to the deeper, anoxic layers.

Notably, the formation of OC-Fe aggregates results in a net gain for the global redox balance at the surface of the Earth as the reduction of one mole of iron consumes one mole of electrons, while the oxidation of one mole of reduced OC liberates on average about 4.48 moles of electrons^27^. Using a conservative estimate for the total amount of OC sequestered in marine sediments via direct bonding to iron oxides as 19 × 10^15^ g of C^7^, this mass of reduced C amounts to the burial of 7.1 × 10^15^ moles of electrons. This value is corrected by also considering the burial of OC-complexed oxidized iron, which would otherwise be reduced via the transfer of one electron per mole of Fe(III). Using the average corrected OC:Fe molar ratio of 5.02 obtained from the slope of Fig. 6, we find that the stabilized Fe that escapes reduction amounts to 3.2 × 10^14^ electrons, giving a total corrected transfer of 6.8 × 10^15^ electrons via this synergistic ferric organic carbon shuttle. Such back-of-the-envelope calculation is an oversimplification of a very complex system but it provides a rough estimate of the importance of this mechanism in the transfer of reducing power from the surface of the Earth to the slowly cycling sedimentary rock carbon pool, contributing to the stability of the global redox balance and the Earth’s oxygenated atmosphere.

The strong inner-sphere complexes formed between OC and Fe(III) in marine sediments play a critical role in the sequestration of organic matter. These interactions are mutually beneficial as they allow for the stabilization and burial of OC, which would otherwise be mineralized back to CO_2_ or CH_4_, while also stabilizing reactive iron in low oxygen environments (such as the Black Sea). Protection of OC and stabilization of reactive iron within sediments allows for these complexes to escape reductive dissolution, which occurs near the sediment oxic/anoxic redox boundary. This protection mechanism allows for both reactive and intrinsically stable organic matter to escape degradation over very long timescales.
